# Performance and Obstacle Tracking to Natural Forest Resource Protection Project: A Rangers’ Case of Qilian Mountain, China

**DOI:** 10.3390/ijerph17165672

**Published:** 2020-08-05

**Authors:** Ya Wang, Lihua Zhou, Guojing Yang, Rui Guo, Cuizhen Xia, Yang Liu

**Affiliations:** 1 Key Laboratory of Desert and Desertification, Northwest Institute of Eco-Environment and Resources, Chinese Academy of Sciences, Lanzhou 730000, China; wangya2014@lzb.ac.cn; 2 Institutes of Science and Development, Chinese Academy of Sciences, Beijing 100190, China; xiacuizhen@lzb.ac.cn (C.X.); liuyang18c@mails.ucas.ac.cn (Y.L.); 3 University of Chinese Academy of Sciences, Beijing 100049, China; guorui@nieer.ac.cn; 4 Key Laboratory of Ecohydrology of Inland River Basin, Northwest Institute of Eco-Environment and Resources, Chinese Academy of Sciences, Lanzhou 730000, China; yangguojing@lzb.ac.cn

**Keywords:** natural forest resource protection project, rangers, performance evaluation, public value, GRA-TOPSIS, Qilian Mountain

## Abstract

Forests play an important role in the process of land degradation and restoration. As a national key ecological project for protecting natural forest, the natural forest resource protection project was implemented in 17 provinces for nearly 20 years. As the core stakeholders and main force for protecting forest resources, rangers have a clear, more objective and comprehensive perception of the policy process, problems and forest ecological changes than farmers. This study introduces public value theory, builds a performance evaluation system that combines the “process-outcome” of ecological construction and uses the GRA-TOPSIS and obstacle tracking model to investigate the performance and obstacle factor of natural forest resource protection project from rangers’ perspective. GRA-TOPSIS is an optimal sequence technique for ideal solution optimization that combines the gray correlation method. The empirical results showed the overall performance of the natural forest resource protection project is good, the relative gray closeness that indicated the process dimension value of the natural forest resource protection project (NFRPP) is 0.663 which higher than the outcome dimension. It reflected the characterization and value level of overall and dimensions performance of NFRPP in Qilian Mountain. The rangers’ support evaluation is the highest, followed by the ecological outcome, sustainability and stability. The key obstacle is the support of local farmers, the social and economic outcome of the project. The natural forest resource protection project has shortcomings in its management system, function setting and support mechanism and urgently improved it from the resource system, resource unit, management system and user. These results are important to promote better implementation of such ecological projects, to enhance the project stability and the regional sustainable development.

## 1. Introduction

Qilian Mountain is an important and typical area of ecological security barrier in western China and ecological construction of the Silk Road Economic Belt. In recent years, the ecological problems such as the glaciers retreat, snow line elevation and water conservation efficiency of mountain forest grassland decreased are outstanding [1,2,3], especially the northern piedmont of Qilian Mountains [4]. The natural forest resource protection project (NFRPP), as a protection policy to guarantee the construction of water conservation forest and protection of natural forest resources in Qilian Mountains, what is the process, outcomes and performance of NFRPP that invest 782 million in the past 20 years? Whether the NFRPP has achieved the expected ecological effect? What about the rationality (whether the implementation is in place, whether NFRPP is efficient, policy fairness, participation and satisfaction of farmers) and sustainability of engineering? How to evaluate the performance?

As the latest development after the four paradigm changes in public administration, public value—which combines democratic values and management, is a sign of the renaissance of the public interest and the balance and soundness of public administration [5,6,7]. Mark Moore believes that the public value is a collection of citizens’ expectations of the government [8]. It is a utility that the public obtained through the practical public policies and services [9]. This method will provide a new perspective for ecological policy evaluation and new system construction, as well provide a new attempt to incorporate ecological construction policies into the government performance management system [10]. To date, the evaluation and balancing of public value no longer stay at the static level but integrates instrumental rationality and value rationality in dynamic and tangible actions [11,12]. The focus of the project performance evaluation should move forward from the static result evaluation to the entire process of project management and covering the two dimensions of the process and results of project management.

Previous studies have focused on the NFRPP’s ecological benefit monitoring and government performance evaluation, livelihood impact (forest enterprises, poverty in the forest area, conversion and re-employment of workers) and forest ecological benefit compensation system, implementation status and problems analysis in typical regional and its subsequent development management measures. These studies only pay attention to single aspect of project ecological effect or participants’ behavior, pursue the highest efficiency of government performance management. Them lack the discussion on the rationality of policy implementation process and its public value (such as fair, just and democratic). As a purely public welfare project which purpose is restoring and rebuilding the ecosystems, ecological engineering has outstanding externalities and involves many relevant stakeholders. The composition and transmission of ecological engineering public value are mainly reflected in the production and provision of ecological services by the government, the public (project participants, local residents and other potential and marginal stakeholders) pursued the values of fairness, efficiency, economical, sustainability and democracy and the value transfer and effect inspection of the result part of ecological engineering [13]. Public value covers the entire process and results, including the goals of public preference identified by governments and the public subjective satisfaction and the implementation process fairness [6]. To date, the performance evaluation of ecological engineering has shifted from outcome-oriented to process-oriented, paying more attention to the mining and pursuit to project’s own public value [10].

Thus, unlike the previous studies, we introduce the public value theory to the study of ecological project performance evaluation. From the rangers’ perspective to analyze the effectiveness and rationality of NFRPP by GRA-TOPSIS method and answer the question of how to continue and adjust in the next stage? We selected the North piedmont of Qilian mountain where are in Northwest China as a NFRPP case study. Our analysis will provide a scientific basis for the timely adjustment of the NFRPP and point out the management countermeasures in the future. The innovation of this study is building a “process-outcome” performance evaluation framework and indicator system that combined the project execution process with outcomes and multiple subjects to evaluate the NFRPP performance.

## 2. Materials and Methods

### 2.1. Overview of the Policy for NFRPP

1950s, the policies of steel smelting and grain-based that advocated by the “Great Leap Forward” led to serious land reclamation and large-scale primary forest destruction in the northern China. With the acceleration of the reform and opening policy, the society demand for wood and other forest products had surged. In order to protect the forest resources and improve the service functions of forest ecosystems, after the Yangtze River Basin floods, the Chinese government had launched a pilot project of NFRPP in 12 provinces which including Yunnan, Chongqing and Gansu. The project contents were mainly to stop commercial logging of natural forests, forest management, mountain closure and barren afforestation. This project is tasked with the multiple goals of forest ecosystem protection, ecological function enhancement and strategic transformation of forest industry enterprises. In December 2000, the ten-year phase of NFRPP was launched with the policy that 80% of central government funding and 20% of local government matching. The scope involved the 17 provinces, 734 counties and 167 forestry bureaus where located in the upper Yangtze River, the upper and middle Yellow River and the Northeast, Inner Mongolia, Hainan and Xinjiang province. In 2011, the total area of the second phase of NFRPP was expanded from 2.63 × 10^8^ hm^2^ to 2.74 × 10^8^ hm^2^ and added the 11 counties in Danjiangkou reservoir of Hubei and Henan province. Compared with the first phase of NFRPP, the second phase is in line with the reform of collective forest rights system, integrating the forest management and ecological compensation policies, greatly increasing the subsidy standards for forest resource protection, ecological public welfare forest construction and government and social expenditures. Consequently, the subsidy for state-owned forests and collective forests rose from 1.75 CNY/ha/year in first phase to 5 and 10 CNY/ha/year, respectively. Simultaneously, a new subsidy policy for young forest tending and cultivation of reserve resources in the state-owned forest zone where located in Northeast, Inner Mongolia, Hainan and Xinjiang province was added and the requirement of 20% of local supporting funds was cancelled.

### 2.2. Overview of the Study Area

**Figure 1:**
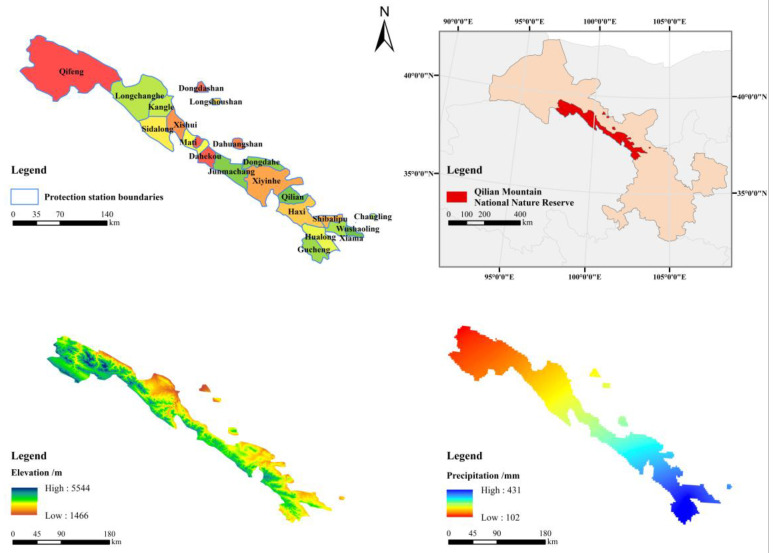
Overview map of Qilian Mountain National Nature Reserve (QMNR). Note: Figure 1 is composed of four maps: the distribution map of the protection stations of Qilian Mountain National Nature Reserve, the geographic location of the nature reserve, the elevation map and the precipitation distribution map.

Qilian Mountain (35°50′ N–39°19′ N, 94°10′ E–103°04′ E) is located at the confluence of the three plateaus of Qinghai-Tibet, Mongolia and Loess in the Northwest of China. It cross the borders of Gansu and Qinghai province and divide the north and south foothills according to the provincial boundaries. The difference in water and heat caused by the different landscape. The northern slope (Gansu) of Qilian Mountain is dominated by shrubs and forests. There are 5.90 × 10^3^ km^2^ of woodland within the Gansu National Nature Reserve of Qilian Mountain (Figure 1), including 1.41 × 10^3^ km^2^ of arbor forest, 1.76 × 10^2^ km^2^ of sparse forest, 4.30 × 10^3^ km^2^ of shrubland, 7.09 km^2^ of undeveloped forest, no stand woodland 4.41 km^2^ and suitable woodland 2.32 km^2^. The coverage rate of arbor forest reaches 6.45%. The trees are basically the natural secondary forest. Qinghai spruce, Qilian juniper, poplar, birch and Chinese pine are the main tree species. The Shrub plants such as golden plum, mountain willow, caragana, azalea are sporadic distribution. Natural forests, as the “lifeline of human homeland”, are the main body of forest resources. It has a very important ecological value in water conservation, soil conservation, carbon fixation and oxygen production and biodiversity protection. It is the natural “green reservoir” in Hexi corridor, the total soil water storage is 5 × 10^8^ m^3^, and the total value of ecosystem service functions is 654.44 × 10^8^ CNY [14].

Qilian Mountain, known as the important ecological barrier of Qinghai-Tibet Plateau and ecological construction of Silk Road Economic Belt, is the birthplace of inland rivers in Hexi corridor. In 2000, QMNR in Gansu province was formally incorporated in the “NFRPP in the upper and middle reaches of the Yellow River”. By 2008, the forest area of water conservation in the northern foothills of Qilian Mountains was listed as 50 important ecological service function areas. The central government had invested 782 million CNY (actual investment of 238.878 million CNY from 2000 to 2010 and planned to invest 53.63754 million CNY from 2011 to 2020) to cease commercial logging of natural forests and closure mountain, afforestation in 22 protection stations of nature reserve. The project of NFRPP effectively improved the ecology and achieved the double growth of forest resources area and accumulation. The area of forested land, sparse forest land, shrub land and undeveloped forest land increased by 4.8%, 26.9%, 54.3% and 283.6% compared with 2000, respectively [14]. The forest coverage rate and standing timber accumulation increased by 1.3%, 212.47 × 10^4^ m^3^ and the annual added value of the forest ecosystem services was 155.82 × 10^8^ CNY [14].

### 2.3. Questionnaire Design and Data Collection

Previous studies have shown that “who will assess, what to assess and how to assess” are the three key issues for scientific and systematic assessment of the benefits of forestry ecological project. The behavior patterns, communication systems and preference choices of relevant stakeholders are the carrier chain for the creation, transfer and realization of public value of ecological project [15,16]. Yang et al. believe that the main body of forestry ecological project for benefit assessment include three types of core stakeholders, potential stakeholders and marginal stakeholders [16]. As the core stakeholders and main force for management and protection of forest resources, rangers are the patrollers and propagandists at the grass-roots level of forestry. They have a clear, objective and comprehensive perception of the process, problems and forest ecological changes in policy implementation. Simultaneously, rangers as the national public officials, there is a stable team with sufficient sample size. Hence, the project group issued a questionnaire titled “Performance evaluation of NFRPP in Qilian Mountains from rangers’ perspective” to learn more information. The questionnaire include social property information of the rangers, protection station organization and management, social–ecological change perceptions of project areas, surrounding farmers’ behavior and attitude towards NFRPP, evaluation, problem and proposal of NFRPP. Details of the questionnaire are shown in Table 1.

**Figure 2:**
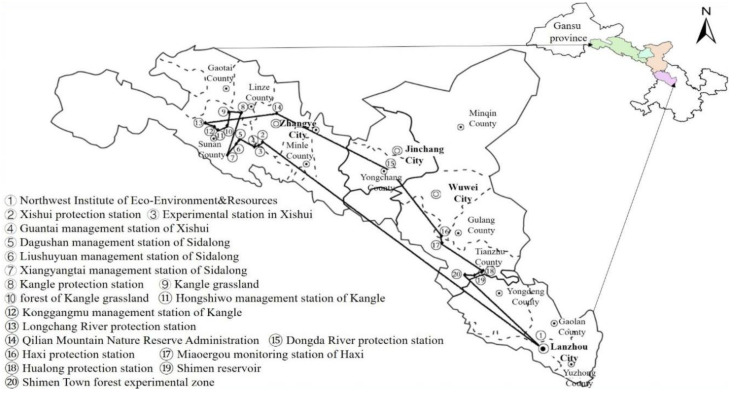
Survey route and site.

Data for this study were obtained from a survey of 42 rangers in the QMNR of Gansu province undertaken in June 2019. We conducted an in-depth investigation on the implementation status and performance of NFRPP in the followed protection station (Figure 2): Xishui (Guantai management station, Taergou forest), Sidalong (Dagushan station, Liushuyuan station, Xiangyangtai station and Tianluochi forest), Kangle (forest of Kangle grassland, Konggangmu station), Longchang River, Dongda River, Haxi (Miaoergou station and its monitoring station), Hualong (Shimen Town forest, Shimen reservoir). This survey included policy copywriting research (the content involved the engineering planning, operation design and mountain log of rangers), job-site project survey (the survey focus contained the NFRPP progress status and forest ecosystem restoration) and the rangers’ investigation. We randomly selected three or four rangers (exclude the ecological rangers who are the local poverty-stricken farmers) in the above station, a total of 42 rangers were selected. The survey time for each questionnaire were about 60 min and the valid questionnaires were 40. The respondents were between the ages of 25 and 55, with an average age of 39; the working years range from 5 to 30 years, with an average year of 19. The minority (Yugu) ethnic group of respondents accounted for 25%, the rest were Han nationality. They have a relatively high level of education, 77.5% of them have junior college education or above. The persons who are in charge of protection stations or subordinate stations accounted for 52.5% of the respondents. They have a comprehensive understanding of the implementation of the NFRPP, more objective answers to the questionnaire. Hence, the credibility of the survey results is higher. Although there were fewer questionnaires, the respondents were more representative and typical.

### 2.4. Framework and Index

#### 2.4.1. Measurement Framework of the Performance for NFRPP

The current performance evaluation methods of ecological engineering are the followed three type: the “ecological–economic–social” benefit evaluation method which is based on the result-oriented, the transaction cost measurement method which is based on the institutional structure, and the balance scorecard performance evaluation tool which is goal-oriented [5]. As the mainstream evaluation method, the value of ecological services is the basis of the results-oriented performance method which establishes an indicator system from the benefit of ecological, economic and social to reflect the result of the project. This method can do a concise result judgment on whether ecological engineering has achieved the ultimate goal. However, it does not consider the process elements of the behavior and satisfaction of the relevant stakeholders (government and farmers) who participate in ecological construction. Therefore, it is difficult to explain the rationality and fairness of ecological engineering performance. The transaction cost theory that based on the new institutional economics is focused on the decomposition and quantification of transaction costs within the engineering policy itself to reveal the project internal mechanism and game process. Its disadvantage is the evaluation results can only reflect the execution of the policy formulation and implementation process on government-level. It cannot express the responsiveness and participation of the other relevant subjects (such as farmers) and even ignore the public value of project. This method is difficult to combine the implementation process of policies with ecological results and the impact of people’s livelihood, resulting in a certain one-sidedness in the evaluation results. The balanced scorecard is a goal-oriented evaluation tool that serves enterprise development strategy. This method transforms the tasks and decisions of the company and its internal departments into diverse goals and then breaks down the goals into multiple dimensions including financial status, customer service, internal business process, learning and growth. Although the domestic scholars have improved this method to four levels of finance, public, internal processes and learning growth and applied it to the performance evaluation of NFRPP, the paradigm is insufficient to explain the effectiveness of ecological engineering, especially whether the project has achieved the expected ecological effect.

**Figure 3:**
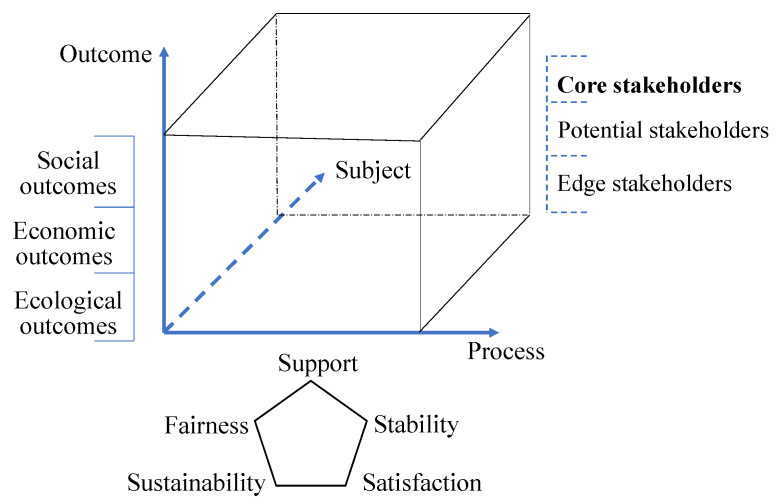
Illustration of the performance evaluation framework.

To overcome the flaw of the above methods which only focus on the engineering results, ignore the implementation process, only focus on the government level and ignore other stakeholders’ responses and lack of reflection on public value, this study introduces the public value theory to take into account the process and outcome dimensions of the aforementioned performance implications. Then, builds a performance evaluation framework that combines the process, outcome and subject of ecological construction (Figure 3) to measure whether the project is achieving the desired goal. The purpose of this method is to provide ideas and case support for the design of the overall optimization plan for the construction of Qilian Mountain National Park and the further adjustment of ecological engineering.

#### 2.4.2. Evaluation Index System of the Performance for NFRPP

Public value is a new research perspective in government performance management which can reflect the “efficiency” of public management, the “fairness” of engineering policies, the respect for the cooperative production subjects and the “sustainability” of policy effects [10,17]. According to the basic provisions of public value and the characteristics of ecological policies, the indicators in process dimension are selected from support, stability, sustainability, satisfaction and fairness (Table 2). The support and cooperation of the participants is the basis for the NFRPP successful development. It needs to be reviewed from the perspective of rangers (the most important practitioners) and local farmers (the project potential participants). The resignation willingness of rangers, the conditions of working environment and climate are the key issues to determine the persistent of NFRPP. Poor working conditions and strong resignation willingness of staff will accelerate the rangers’ mobility, which will bring the risks of manpower shortage, excessive management area per capita and insufficient care. As a merit project that benefits people’s livelihood, diversion, placement and social security construction for forestry staff is the important part of NFRPP. For rangers, the fairness of NFRPP is mainly reflected in the forest management subsidies. It can be characterized by subsidies satisfaction. The rangers’ perception of the development prospect of forest area and the demand for NFRPP sustainability under the background of ecological restoration in Qilian Mountain can reflect the potential and sustainability of NFRPP. Engineering satisfaction can be decomposed into the perception of engineering and job satisfaction. The outcomes of NFRPP can be evaluated by the rangers’ intuitive perception from the ecological, social and economic benefits of the project. According to the average statistics of indicators, rangers have the highest evaluation of working environment B1(4.15) and the lowest evaluation of local farmers’ support to NFRPP A2 (2.38) in process dimension. While the social outcome evaluation F3 is higher (0.108), and the ecological outcome F2 is lower (0.085) in outcome dimension. The highest weight is the index of farmers’ support to NFRPP A2 (0.190), the lowest weight is the index of NFRPP ecological require C2 (0.007). The calculation steps of the index weight are detailed in the Appendix A.

### 2.5. Models Selection

#### 2.5.1. The Improved TOPSIS Model Based on the Gray Relation Analysis

Technique for Order Preference by Similarity to Ideal Solution (TOPSIS) is a management decision method proposed by C.L. Hwang and K. Yoon (1981) for multidimensional vector comparison and ranking, also known as the pros and cons solution distance method or double basis point method [18]. Euclidean distance calculation is the core of TOPSIS. It is derived from the true distance between two points in the Euclidean m-dimensional space to describe the differences between the two objects. TOPSIS constructs a normalized multi-attribute matrix to detect the Euclidean distance between the object and ideal solutions (including the positive and negative), calculates the relative closeness of each attribute to its idealized target to diagnose the ranking of the measured objects. This method has no special requirements for sample data, the calculation is flexible and simple. It can systematically analyze the gap between the object and the ideal state to truly reflect the problems and provide the direct and clear decision information to decision-makers [19]. Based on the above advantages, this method is widely used in the field of rural revitalization and ecological civilization construction on the performance evaluation of land use, rural environmental improvement, poverty alleviation, ecological engineering and policy, resource carrying capacity and the vulnerability and safety social–ecological system [20,21,22]. However, the TOPSIS model can only reflect the positional relationship because it uses Euclidean distance to measure the pros and cons of each object. It is difficult to express the difference in the change trend of each object and reflect the situation changes among data series of objects. In view of the above deficiencies of TOPSIS, this study introduced the gray relation analysis (GRA) method that proposed by Deng Julong to improve it. The essence of GRA is to determine the closeness of the connection between different sequences by the similarity of the geometric shape of the data sequence curve. It can reflect the close sequence of the comparison sequence to the reference sequence to better explain the fuzziness association and change trend of the object internal factors. Combining the gray correlation with TOPSIS can realize the standardization of positive and negative ideal distances and gray correlation degree and finally achieve the purpose of correcting the relative proximity [15]. The specific steps of the improved method are as follows:

Step 1: Construct a weighted standardized matrix. The index weights that obtained by the entropy weight method (Appendix A) are multiplied with standardized data one-by-one to construct a weighted standardized matrix Z *. Reference the Equation (1).

(1) $$Z*={\left(z_{ij}\right)}_{m\times n}=\left[\begin{array}{cccc} w_{1}r_{11} & w_{2}r_{12} & \cdots & w_{n}r_{1n}\\ w_{1}r_{21} & w_{2}r_{22} & \cdots & w_{n}r_{2n}\\ \vdots & \vdots & \vdots & \vdots \\ w_{1}r_{m1} & w_{2}r_{m2} & \cdots & w_{n}r_{mn} \end{array}\right]$$

where *w* represents the weight of each index, *r* represents the standardized data.

Step 2: Determine the ideal solutions. According to Z *, determine the positive ideal value and negative ideal value of each indicator. Reference the Equations (2) and (3).

(2) $$V^{+}=\left\{\underset{1\leq i\leq n}{\max Z_{ij}}|i=1,2,\cdots ,n\right\}=\left\{V_{1}^{+},V_{2}^{+},\cdots ,V_{m}^{+}\right\}$$

(3) $$V^{-}=\left\{\underset{1\leq i\leq n}{\max Z_{ij}}|i=1,2,\cdots ,n\right\}=\left\{V_{1}^{-},V_{2}^{-},\cdots ,V_{m}^{-}\right\}$$

where *V*^+^ represents the maximum value of the *i*–th indicator to j-ranger, it is the most ideal solution and named the positive ideal solution. *V*^−^ represents the minimum value of the *i*–th indicator to j-ranger, it is the worst solution and named the negative ideal solution.

Step 3: Calculate the Euclidean distance of $D_{i}^{+}$ and $D_{i}^{-}$ between the index value of each sample and the positive and negative ideal solutions (Equations (4) and (5)).

(4) $$d_{i}^{+}=\sqrt{\sum_{j=1}^{m}{\left(V_{ij}-V_{j}^{+}\right)}^{2}}\left(i=1,2,\cdots ,n\right)$$

(5) $$d_{i}^{-}=\sqrt{\sum_{j=1}^{m}{\left(V_{ij}-V_{j}^{-}\right)}^{2}}\left(i=1,2,\cdots ,n\right)$$

Step 4: Calculate the gray correlation coefficient. $\xi_{ij}^{+}$ is the gray correlation coefficient between each indicator and the positive ideal set (Equation (6)). $\xi_{ij}^{-}$ is the gray correlation coefficient between each indicator and the negative ideal set (Equation (7)). Ρ∈ [0,1] is the resolution coefficient, generally takes the value 0.5.

(6) $$\xi_{ij}^{+}=\frac{\underset{i}{\min}\underset{j}{\min}\left|V_{j}^{+}-V_{ij}\right|+\rho \underset{i}{\max}\underset{j}{\max}\left|V_{j}^{+}-V_{ij}\right|}{\left|V_{j}^{+}-V_{ij}\right|+\rho \underset{i}{\max}\underset{j}{\max}\left|V_{j}^{+}-V_{ij}\right|}$$

(7) $$\xi_{ij}^{-}=\frac{\underset{i}{\min}\underset{j}{\min}\left|V_{j}^{-}-V_{ij}\right|+\rho \underset{i}{\max}\underset{j}{\max}\left|V_{j}^{-}-V_{ij}\right|}{\left|V_{j}^{-}-V_{ij}\right|+\rho \underset{i}{\max}\underset{j}{\max}\left|V_{j}^{-}-V_{ij}\right|}$$

Step 5: Calculate the closeness of positive and negative ideal. Using the Equations (8) and (9) to calculate separately the gray correlation $g_{i}^{+}$ and $g_{i}^{-}$ between sample j and the ideal solution.

(8) $$g_{i}^{+}=\frac{1}{n}\sum_{j=1}^{n}\xi_{ij}^{+}\left(i=1,2,\cdots ,n\right)$$

(9) $$g_{i}^{-}=\frac{1}{n}\sum_{j=1}^{n}\xi_{ij}^{-}\left(i=1,2,\cdots ,n\right)$$

Step 6: In order to overcome the dimensional difference between different data, the Euclidean distance and gray correlation degree of positive and negative ideal solutions are dimensionlessly processed using Equation (10).

(10) $$D_{gi}^{+}=\frac{d_{i}^{+}}{\underset{i}{\max d_{i}^{+}}}D_{gi}^{-}=\frac{d_{i}^{-}}{\underset{i}{\max d_{i}^{-}}}G_{gi}^{+}=\frac{g_{i}^{+}}{\underset{i}{\max}g_{i}^{+}}G_{gi}^{-}=\frac{g_{i}^{-}}{\underset{i}{\max}g_{i}^{-}}$$

Finally, the Euclidean distance and gray correlation degree standardized merged by the Equation (11) [23] and the calculated the relative closeness. Refer to the performance evaluation results of the previous research fruit, we divided *C_i_* into four levels: defined the values (0.80, 1.00) as excellent, (0.60, 0.80) as well, (0.30, 0.60) as medium and [0, 0.30] as poor.

(11) $$C_{i}=\frac{\beta_{1}D_{gi}^{-}+\beta_{2}G_{gi}^{+}}{\left(\beta_{1}D_{gi}^{-}+\beta_{2}G_{gi}^{+}\right)+\left(\beta_{1}D_{gi}^{+}+\beta_{2}G_{gi}^{-}\right)}$$

where *C_i_* represents the relative closeness with the *j*-th ranger to optimal solution, its value is larger explained the object to be evaluated is better. *Β*_1_ is the preference coefficient, which can reflect the decision-maker’s preference for *G_gi_* (curve shape) and *D_gi_* (Euclidean distance). Here, both *β*_1_ and *β*_2_ take the value 0.5 according to the study of Ren et.al.

#### 2.5.2. Obstacle Factor Tracking Model

To identify the main obstacle factors that impede the NFRPP performance to adjust the current development planning and policy regime. Using the “obstacle factor model” (Equation (12)) to conduct a pathologic diagnosis. Scholars have applied this method to track the key factor and understand the drivers of land use change [24,25], water poverty [26,27] and the ecological structure identification [28].

(12) $$M_{i}=\frac{\left(1-X\prime_{ij}\right)\times W_{i}}{\sum_{i=1}^{13}\left[\left(1-X\prime_{ij}\right)\times Wi\right]}\times 100\%$$

where *M_i_* is the obstacle score of the individual indicator. (1 − *X’_ij_*) indicates the index deviation, it is the difference between the individual indicator and its optimal target, i.e., the difference between the individual indicator normalized value and 100%. *W_i_* represents the contributing factors that is the degree of influence of a single factor on the overall objective, i.e., the weight.

## 3. Results

### 3.1. GRA-TOPSIS Results for Performance Evaluation of NFRPP

#### 3.1.1. The Score and Distribution of Performance Value in Process Dimension

**Figure 4:**
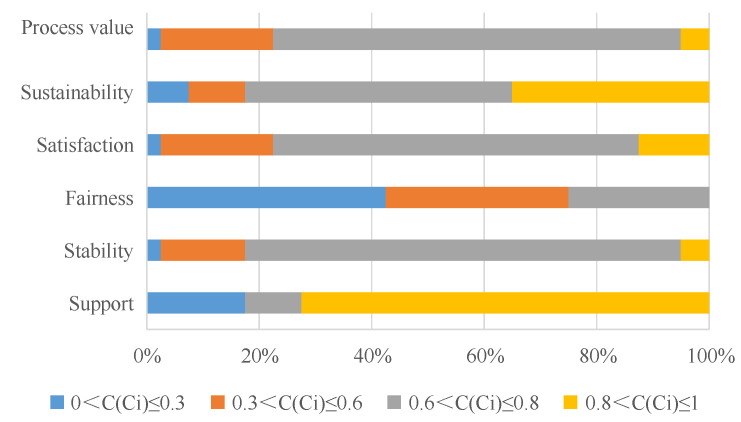
Distribution of performance value of process dimension in different interval. Note: *Ci* indicates the relative gray closeness of each component. C indicates the relative gray closeness of process dimension.

**Figure 5:**
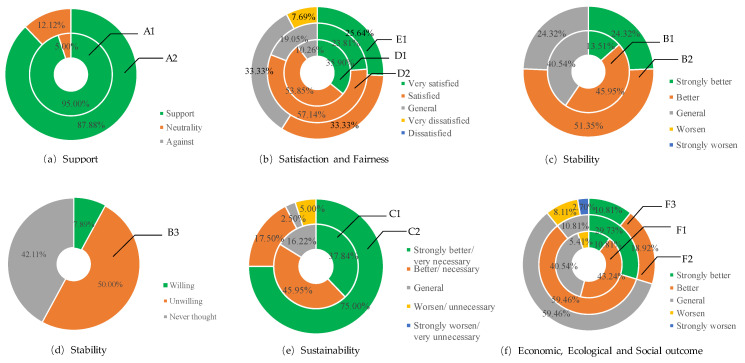
Frequency distribution of rangers’ evaluations on each indicator. (**a**) indicate that the rangers’ perception on NFRPP support. (**b**) indicate that the satisfaction and fairness of NFRPP. (**c**,) and (**d**) indicate that the stability of NFRPP. (**e**) indicate that the sustainability of NFRPP. (**f**) indicate that the NFRPP outcome of economic, ecological and social. The indicator code has the same meaning as Table 1.

According to the GRA-TOPSIS results, the process value (average of *C*) of NFRPP is 0.663 (represents the relative closeness with the total rangers to optimal solution is larger, it explained the object to be evaluated is better) and the gray correlation ($\xi_{ij}^{+}$) and closeness of the positive ideal solution ($G_{i}^{+}$) are 0.795 and 0.813, respectively. Of the ranger’s process performance value, 72.5% is between (0.6, 0.8], only 5% is greater than 0.8. It indicated that the total performance of NFRPP in process dimension is good. The support has the highest score among process components. The average gray closeness of support is 0.732 and the gray correlation and closeness with the positive ideal solution are the largest (Table 3). In frequency distribution, 72.5% of ranger’s support evaluation is higher than 0.8, only 17.5% assessments are below 0.3 (Figure 4). With the introduction of social capital and community co-management systems, forest certification, carbon sink trading, ant forest and other multichannel financing methods have become an important way to solve the funding gaps of government’s public financial system. Therefore, the rangers who hold a neutral attitude toward the support of NFRPP is accounted for 5% (Figure 5a). For local farmers, 87.88% of rangers believed they still support the NFRPP (Figure 5a). Meanwhile, the residents’ livelihoods of the Qilian Mountain water conservation area are highly dependent on forest and grass resources. Although the farmers have a high degree of support for NFRPP, overloading and trespass grazing are common in their actual action’s presence. In the QMNR, there are about 69.4 × 10^4^ hm^2^ shrub forest and 1.9 × 10^4^ hm^2^ sparse forest land with both forest and grassland right certification [4]. “One site and two certificates” objectively provides reasonable conditions for ecological destruction.

The score of sustainability and stability is ranked second and third in process dimension. The average gray closeness of them is 0.678 and 0.663. The frequency ratio with a score less than 0.6 are accounted for 17.5%, which is between (0.6,0.8], the proportion of stability and sustainability is 77.5% and 47.5%, respectively. Even, 35% of the rangers ’sustainability evaluation is greater than 0.8, it indicates the Qilian Mountain ecological restoration have a high demand for the NFRPP. Among the respondents, 57.14% of the rangers are satisfied with the current job (Figure 5b), and 45.95% of the rangers believe the working environment is better (Figure 5c). Due to the work habits and home in the forest area, 50% of rangers are reluctant to work outside the forest (Figure 5d). Only 7.89% of rangers consider changing jobs because of the difficult conditions in the forest area and they cannot take care of the elderly and children (Figure 5d). As an attempt to solve the cross-regional and cross-departmental institutional problems, the Qilian Mountain National Park System Pilot was launched in June 2017. What are the prospects of NFRPP under the above background? The respondents generally believe that natural forest is an important carrier to maintain national ecological security and respond to climate change. 75% of the rangers believed the NFRPP is very necessary for the ecological restoration sustainable of Qilian Mountains (Figure 5e). About 37.84% of rangers perceived the construction of national parks is an important guarantee for the lasting effects of NFRPP. They expected the State Council to increase the investment and continue to carry out the ecological construction such as logging prohibition, forest management, closure and afforestation. In short, exploring the balances point between ecological protection and well-being development will be the key issues for NFRPP.

It is worth noting that the average distance, gray correlation and closeness of the satisfaction score with the worst solution are the highest scores among the five process elements, which are 0.803, 0.551 and 0.409. In terms of frequency distribution, 12.5% of rangers’ satisfaction score is greater than 0.8, 22.5% is less than 0.6 and 65% is between (0.6, 0.8]. It indicated that the rangers’ satisfaction with NFRPP is high. Among the respondents, 89.74% of the rangers are satisfied (53.85%) or very satisfied (35.90%) with the current NFRPP (Figure 5b). They believed the forest and woodland had grown; the forest area ecological had be improved after NFRPP. The mountains closure and barren afforestation had increased the forest area within the QMNR by 44.0%, the standing tree stock had increased by 212.47 × 10^4^ m^3^, the value of forest ecosystem services is 155.82 × 10^8^ CNY/year [14]. Of the rangers, 57.14% were satisfied with the current job because of the relatively stable salary income and social security (Figure 5b). The state had greatly increased the employees’ salary in the second phase of NFRPP. Compared with the first phase, the salaries of rangers have increased significantly. Set Haxi protection station as a case, the average salary of staff on duty reached 90,488 CNY in 2018, which was higher than the average social income. 

Among the five process elements, the fairness score is the lowest (the average gray closeness is 0.315) and the gray closeness to the positive ideal solution is the lowest (0.139). A total of 42.5% and 32.5% of the rangers rated the NFRPP fairness as “poor” or “medium”, only 25% of the rangers’ value was in (0.6, 0.8]. In the field survey, 7.69% of the rangers did not satisfied with the current subsidy standard for forest resource management (Figure 5b). They believe that the natural resources protection funds of nature reserve is relatively low. There is unfairness in the sharing of financial mechanisms and funds allocation. This unfairness is mainly manifested in: (1) less investment in forest fire prevention and forest pest control; (2) lack of special funds for infrastructure construction and maintenance of forest areas such as roads, houses and electricity; (3) no special funds for cultivating continuous industries in the project area. With the expansion of afforestation scale, afforestation land is transferred to the places where with inconvenient transportation and poor site conditions, which makes afforestation more difficult and increases costs. Hence, 77.5% and 62.5% of rangers expect the state to increase the standard of subsidies and extend the period of subsidies.

#### 3.1.2. The Score and Distribution of Performance Value in Outcome Dimension

**Figure 6:**
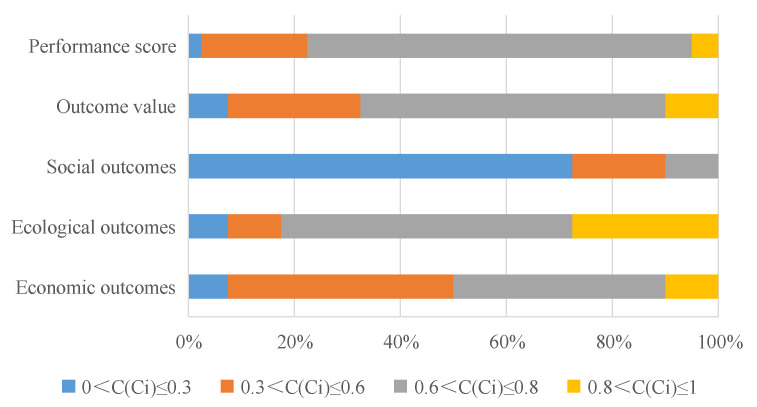
Distribution of performance value of outcome dimension in different interval. Note: *Ci* indicates the relative gray closeness of each component. C indicates the relative gray closeness of outcome dimension and the overall performance.

According to the GRA-TOPSIS results, the outcome value (average of *C*) of NFRPP is 0.621 and the gray correlation and closeness of the positive ideal solution are 0.663 and 0.664, respectively. A total of 57.5% of ranger’s outcome performance value is between (0.6, 0.8], only 10% is greater than 0.8. It indicated that the rangers’ evaluation results are inconsistent. The total performance of NFRPP in outcome dimension is good. The ecological outcome has the highest score among the outcome components (average gray closeness is 0.701), the gray correlation and closeness with the positive ideal solution are the largest (Table 3). In frequency distribution, 27.5% of ranger’s ecological outcome score is higher than 0.8, only 17.5% assessments are below 0.6 (Figure 6). While, the social outcome score is lowest, the average gray closeness is 0.213, the gray correlation and closeness with the positive ideal solution are the lowest and 72.5% ranger’s evaluation is less than 0.3. This is because the natural forest in the protected area is prohibited logging, the forest resource consumption has decreased significantly after implementation NFRPP. Compared with 2000, the area of forested land, sparse forest land, shrub land and undeveloped forest land increased by 4.8%, 26.9%, 54.3% and 283.6%, respectively [14]. Hence, the ecological benefits of NFRPP is significant. Although the NFRPP has transformed the forestry economy and the improved the forestry staff income and social security, it has defects in the financial sharing mechanisms and funds ratio. It lacks the special projects funds in road infrastructure construction and maintenance. In particular, with the closure of small hydropower and illegal mines after the ecological rectification in Qilian Mountains, the original subject who bear the obligation to maintain the road had disappeared. Some forest roads surface are bumpy due to lack of maintenance. The fire-resistant access road cannot be maintained and repaired in time, which causes many hidden safety hazards in patrols. Road problems bring many inconveniences to the work and life of rangers, about 10.81% of rangers believe the current traffic situation is worse (Figure 5f). So, the social outcome with forest traffic as a guide is lowest in performance evaluation. Combining the results of process and outcome dimension, the total performance of NFRPP is good and 72.5% of rangers’ performance value is between (0.6, 0.8].

### 3.2. Obstacle Tracking Results of NFRPP Performance

The results of the obstacle factor model showed the farmers’ support to NFRPP, the social and economic outcome value are the key obstacle factors affect the NFRPP performance. Among them, the largest obstacle degree is the farmers’ support to NFRPP. This index’s average obstacle degree is 18.96%, the skewness is 1.37, the kurtosis is 1.63 (Table 4). The contradiction of Qilian Mountain water conservation area which between the inhabitant livelihood and ecological carrying capacity is prominent. The livestock overload is serious and grazing in forest areas is hard to ban. The ecological awareness and protection behavior of farmers have become the key to ensure the NFRPP’s effectiveness continued. Second, the traditional management model of ranger who are the protected areas workers is restricted by authority of protected area management agency, ecological protection conflicts with local interests, and the contradictions between forest and animal husbandry are prominent. In addition, the per capita management area of workers in the protected area is too large (above 660 hm^2^), the protected areas management is becoming increasingly difficult. To alleviate the problems of ecological pressure and rangers’ heavy tasks and promote the community co-management, the local government implemented the ecological migration and selected the poverty household among the immigrants in surrounding villages as an ecological ranger. Ecological rangers have increasingly become an important force in managing forest resources.

The second obstacle factor is the social outcome of NFRPP. Its average obstacle degree is 10.97%, the skewness is 0.33, the kurtosis is 0.80 (Table 4). As an accelerator of economic growth, transportation infrastructure has significant externalities in reducing poverty and improving well-being. Qiu et al. (2017) research shows that the traffic density increased 1%, correspondingly, the per capita net income of rural residents increased by 1.341% [29]. To date, the density of the forestry professional road network in the QMNR is low, and the fire emergency roads cannot be repaired in time. The road conditions are uneven, some road sections are still low-grade gravel roads which have problems such as road surface collapse and potholes. This will undoubtedly not meet the needs of the forest area ecological construction, also brings great inconvenience to the fire prevention, forest patrol and rangers’ life. According to the rangers’ survey, transportation inconvenience is the crux for some rangers who want to resign. Before 1998, the road maintenance funds were drawn from the afforestation fund of timber production. After the NFRPP implement, the forestry changed from industrial type to ecological type. The road maintenance funds could not execute, the local small mines were responsible for the road management. However, a large number of illegally constructed small mines were shut down under the ecological rectification of Qilian Mountain in 2017, the road maintenance lost the responsible body.

The third obstacle factor is the economic outcome of NFRPP. Its average obstacle degree is 9.58%, the skewness is 0.64, the kurtosis is 0.44 (Table 4). The value orientations of economic and environmental benefits which derived the contradiction between environmental protection and economic development. However, in fact, environmental protection and economic development present a contradictory and unified relationship. The value orientation and harmonious development of protection and development is the paradox of the ecological problems of Qilian Mountains. The key to the solution is to clarify the boundary, conversion relationship and tradeoff criteria between ecological protection, people’s well-being and economic development. Only in this way can we find the key check point and realization paths for ecological protection and people’s well-being development. Natural resources are the basis and conditions for economic development. Economic development must protect the environment that is the essential requirement of economic development and the strategic goal of sustainable development. Therefore, the follow-up industry cultivation and the ecological industry chain reconstruction of NFRPP have become the important obstacles affecting its performance.

The obstacles degree of management station’s working environment quality, climate suitability, forest area development prospects, ranger’s resign willingness and ecological restoration effect sustainability were ranked four to seven. This result showed that improving the working environment and infrastructure conditions in forest area, cultivating the follow-up ecological industry, increasing the rangers’ income are conducive to enhancing the generation and transmission of public value of NFRPP. The remaining indicators hinder the creation of the public value of NFRPP is generally low, but we must focus on improving the efficiency and subsidy standards of the government implementation to reduce the obstacles that affect the policy sustainability.

## 4. Discussion

### 4.1. Reflection on the NFRPP

#### 4.1.1. Function Setting in Management System: Multi-Sectoral Management Trigger System Fragmentation

According to the survey, 78.95% of rangers believe the government will continue to play a major role in protecting the local natural forests, 18.42% and 2.63% of rangers think the local farmers and enterprises will become the leading forces. The rationality of government management system setting, and the effectiveness of administrative authority are the keys to NFRPP’s effectively and sustainability. However, NFRPP is implemented under the dual jurisdiction of the provincial forestry authority and local government in practice. For example, the NFRPP in Zhangye City is implemented by the QMNRA that is a provincial unit, while in Wuwei City it is implemented by the Forest and Grass Bureau that is a local department. The subordinate protection stations of QMNR also have the nature of local government state-owned forest farms, which can be said to be a staff group of two management systems. Hence, the decentralized management system lacks a unified task planning and coordination mechanism. It can result in the followed problems: policy conflicts, overlapping policies, double subsidies, disjointed powers and responsibilities and decentralized regulatory forces in the formulation and execution of projects [30].

#### 4.1.2. Implementation Deviation in Management System: Differentiated Logical Direction of Ecological Governance and Goal Conflicts Trigger System Failure

According to the principal–agent principle, NFRPP’s implementation is based on the agency management model which is the central government multi-level entrusted [31]. The central government assigned the task to the provincial government. Both of them followed the political logic in the NFRPP implementation and aimed at natural forest management and ecological restoration. Then the provincial government issued the tasks to local governments and their competent departments. In general, the local government pays more attention to the growth of regional GDP, it follows the work-orientation principle that is administrative logic. The QMNRA, as the main body for putting into effect the NFRPP, mainly follows the governance logic [32]. When the administrative logic and governance logic deviate, the grassroots practice of NFRPP will be discounted. Hence, the differentiated ecological governance logic direction and goal conflicts of different levels of government have led to the unfavorable forestry management system. In particular, after the pilot of the national park system, the chaos in management system became more prominent. It will cause the problem of information asymmetry in the management process and leading to the failure of the constraint mechanism of ecological construction and natural resource management, falling into the “tragedy of political commons” [31].

#### 4.1.3. Support Mechanism Vacancy in Management System: Social Capital Investment and Community Co-Management

The governance philosophy of multicenter governance and community autonomy what proposed by Elinor Ostrom [33] as the third natural resource governance method after the government control and privatization of property rights started to rise globally. Community co-management has become a highly regarded management type for Qilian Mountains ecological management. To solve the capital needs of NFRPP and the insufficient of financial supply, the state encourages social capital and multichannel financing methods such as forest certification and carbon sink transactions. Meanwhile, the state’s guidance for it is still in a written text, lacking the corresponding legal basis and a clear support mechanism at present. Even, in the absence of a corresponding legal basis, the regionalization and jurisdiction of environmental justice have already been used in practice [34].

### 4.2. The Multidimensional Strategies Proposals for NFRPP

The multidimensional strategies proposals are the guarantee for the long-term functioning of the system to achieve sustainable development. Put forward the development proposal of NFRPP from resource system, resource unit, management system and user that based on the Ostrom’s research paradigm of social–ecological systems [33]. In the future, it is necessary to clarify the ecological hydrological mechanism and technical standards for maximizing ecological benefits of vegetation restoration and reconstruction in resource systems. For the resource units, which characterized by forest trees, first, a reasonable layout model of artificial afforestation and theoretical thresholds should be constructed by state; second, moderated thinned and selective thinned the artificial forests with enclosure periods of more than 30 years [2]. The management system should integrate the authority within the pilot area of the unified Qilian Mountain National Park system as soon as possible, establish an information disclosure mechanism and an auditing and accountability system of natural resources assets for leaders’ leaving, encourage and introduce social capital and accelerate the regional application of the community co-management mechanism and ecological poverty alleviation model [35]. With the establishment of Qilian Mountain National Park, the resources users transformed from the single subject of local farmers to the diversified subject (tourists, enterprises and researchers). Carry out ecological protection publicity and education through multiple channels, strict enforcement and strengthen management is the key.

## 5. Conclusions

To overcome the shortcomings of the lack of response of the followed three traditional methods to public value (the “ecological–economic–social” benefit evaluation only emphasize the project results and ignore its process, the balanced scorecard failed to explain the ecological project’s effectively and the transaction cost method only focuses on the government level and ignores the response from other relevant stakeholders), this study introduces public value theory and builds a performance evaluation index system that combines the “process-outcome” of ecological construction. Based on the perspective of rangers who are the management and practitioners, the GRA-TOPSIS and obstacle factor model were applied in the performance evaluation and obstacle tracking from support, stability, sustainability, satisfaction, fairness and ecological, economic and social results of NFRPP. The results show that the overall performance of NFRPP is good, the process dimension performance value is 0.663 higher than the outcome dimension. From the six components that make up the performance evaluation index system of NFRPP, the rangers’ support to NFRPP is the highest, followed by the ecological outcome, sustainability and stability. Among the indicators, the support of local farmers to NFRPP, the social and economic outcome of NFRPP are the key obstacles affecting its performance. In the current management system, the followed problems should be addressed and resolved quickly. They are multi-sectoral management, overlapping policies, double subsidies, chaotic and decentralization management system, the social support mechanisms vacancy for capital investment and community co-management. The government can try to integrate the authority within the pilot area of Qilian Mountain National Park System, promote multi-institutional integration, establish an auditing and accountability system of natural resources assets for leaders’ leaving.

## Figures and Tables

**Table 1:** Details of the questionnaire.

Survey Content	Number	Specific Content
Social attribute information	8	Years of working, education, unit, age, resignation intention and reasons, wage and satisfaction
Protection station organization and management	8	Staff allocation and mobility, management appraisal system, content and conflict of manage work, subsidy recognition and expectation
Social-ecological perception	8	Evaluation the economic, ecological, climate, transportation, working environment, employment mechanism, democratic autonomy, development prospects
Surrounding farmers’ behavior and attitude	6	Awareness protection and vandalism of farmers, the degree of support and satisfaction to NFRPP
Evaluation, problem and advise for NFRPP	7	Comparison of problem and solution in two stage, support, satisfaction, rationality of NFRPP, proposal and prospect

**Table 2:** Index system to evaluate the performance of natural forest resource protection project (NFRPP).

Dimension	Component	Indicators	Assignment	Weight	Mean (std. dev)
Process value	Support	A1: What is your attitude towards NFRPP?	1 = Against, 2 = Neutrality, 3 = Support	0.049	2.85 (0.65)
		A2: What is the attitude of local farmers to NFRPP?		0.190	2.38 (1.13)
	Stability	B1: How is the working environment?	1 = strongly worsen to 5 = strongly better	0.090	4.15 (0.91)
		B2: How is the climate in your forest area?		0.089	3.83 (0.86)
		B3: Are you willing to leave the forest area to work outside?	1 = Willing, 2 = Never thought, 3 = Unwilling	0.085	3.68 (1.08)
	Sustainability	C1: What is the development prospect of your forest area?	1 = strongly worsen to 5 = strongly better	0.088	3.33 (1.19)
		C2: How much does the ecological restoration of Qilian Mountain require for the sustainability of the NFRPP?	1 = very unnecessary to 5 = very necessary	0.007	3.88 (1.25)
	Satisfaction	D1: Are you satisfied with the current NFRPP?	1 = very dissatisfied to 5 = very satisfied	0.035	3.03 (1.19)
		D2: Are you currently satisfied with the job of a forester?		0.026	3.45 (1.18)
	Fairness	E1: Are you satisfied with the subsidy of NFRPP?		0.053	3.70 (1.25)
Outcome value	Economic outcomes	F1: How is the current economic development of your forest area?	1 = strongly worsen to 5 = strongly better	0.096	2.30 (0.81)
	Ecological outcomes	F2: How is the current ecological environment?		0.085	3.90 (1.30)
	Social outcome	F3: How is the current traffic situation?		0.108	4.10 (0.49)

**Table 3:** The evaluation results of the GRA-TOPSIS in process and outcome dimension.

Component	$D_{i}^{+}$	$D_{i}^{-}$	$\xi_{ij}^{+}$	$\xi_{ij}^{-}$	$G_{i}^{+}$	$G_{i}^{-}$	C (Ci)		
							Mean	Max	Min
Support	0.205	0.792	0.905	0.413	0.848	0.309	0.732	0.857	0.143
Stability	0.314	0.752	0.713	0.437	0.733	0.375	0.663	0.857	0.143
Fairness	0.265	0.735	0.705	0.423	0.139	0.344	0.315	0.600	0.000
Satisfaction	0.267	0.803	0.708	0.551	0.756	0.409	0.649	0.828	0.208
Sustainability	0.225	0.780	0.668	0.466	0.724	0.345	0.678	0.857	0.143
Process value	0.327	0.832	0.795	0.512	0.813	0.420	0.663	0.808	0.231
Economic outcomes	0.335	0.665	0.642	0.460	0.653	0.398	0.622	0.857	0.143
Ecological outcomes	0.225	0.775	0.748	0.424	0.762	0.324	0.701	0.857	0.143
Social outcome	0.395	0.605	0.599	0.485	0.100	0.440	0.213	0.600	0.000
Outcome value	0.358	0.665	0.663	0.456	0.664	0.407	0.621	0.857	0.143

Note: $D_{i}^{+}$ indicates the optimal solution distance, $D_{i}^{-}$ indicates the worst solution distance, $\xi_{ij}^{+}$ represents the gray correlation coefficient of positive ideal solution, $\xi_{ij}^{-}$ represents the gray correlation coefficient of negative ideal solution, $G_{i}^{+}$ is the positive ideal solution closeness, $G_{i}^{-}$ is the negative ideal solution closeness. The above values are the average of each dimension. *Ci* indicates the relative gray closeness of each component. C indicates the relative gray closeness of process and outcome dimension.

**Table 4:** Statistical characters of obstacle amounts against the performance of NFRPP.

Index	Mean/%	Maximum/%	Minimum/%	Coefficient of Variation	Skewness	Kurtosis
A1	4.89	4.93	4.82	0.004	−1.19	3.80
A2	18.96	19.42	18.59	0.001	1.37	1.63
B1	8.98	9.10	8.91	0.004	0.48	−0.06
B2	8.88	8.99	8.78	0.005	0.18	0.65
B3	8.48	8.70	8.31	0.009	0.33	0.22
C1	8.78	8.89	8.68	0.006	−0.18	−0.33
C2	0.70	0.70	0.69	0.005	−1.45	2.41
D1	3.49	3.52	3.43	0.005	−1.63	4.86
D2	2.59	2.63	2.54	0.008	−0.59	0.67
E1	5.39	5.53	5.27	0.009	0.05	1.11
F1	9.58	9.70	9.49	0.005	0.67	0.44
F2	8.48	8.59	8.43	0.004	0.57	1.37
F3	10.78	10.97	10.65	0.006	0.33	0.80

Note: indicator code has the same meaning as Table 1.

## Abbreviations

The following abbreviations are used in this manuscript:

NFRPP	Natural forest resource protection project
QMNR	Qilian Mountain Nature Reserve
QMNRA	Qilian Mountain Nature Reserve Administration

## Appendix A

In order to eliminate the influence of each measurement index dimensionally inconsistent on the diagnosis of NFRPP performance, using the extreme value standardization (Equation (A1)) to normalize data. Previous studies mostly used the subjective weighting method of Analytic Hierarchy Process (AHP) which is susceptible to interference from artificial ideas and experience. Hence, this study used the entropy method (Equations (A2) and (A3)) which is an objective weighting method relied on the variation information entropy between data to measure weight. Since the value of “0” will appear after data normalization, the linear interpolation method is used to avoid the occurrence of ln0, so the normalized value is replaced with 0.00001.

(A1) $$r_{ij}=\frac{a_{ij}}{\sqrt{\sum_{j=1}^{40}a_{ij^{2}}}}$$

where *r_ij_* is the standardized value, *a_ij_* is the initial value of *I* performance index to *J* ranger.

(A2) $$e_{i}=-\frac{1}{\ln m}\sum_{i=1}^{m}{\overset{-}{r}}_{ij}\ln{\overset{-}{r}}_{ij}$$

where *e_i_* is the information entropy value to *I* performance index, *m* is the total number of rangers.

(A3) $$W_{i}=\frac{1-e_{i}}{\sum_{j=1}^{13}\left(1-e_{i}\right)}$$

where *W_i_* is the weight to *I* performance index, *n* is the total number of indices, equal to 13.
